# Dutch GP healthcare consumption in COVID-19 heterogeneous regions: an interregional time-series approach in 2020–2021

**DOI:** 10.3399/BJGPO.2023.0121

**Published:** 2024-05-29

**Authors:** Maarten Homburg, Marjolein Berger, Matthijs Berends, Eline Meijer, Thijmen Kupers, Lotte Ramerman, Corinne Rijpkema, Evelien de Schepper, Tim olde Hartman, Jean Muris, Robert Verheij, Lilian Peters

**Affiliations:** 1 Department of Primary and Long-term Care, University Medical Center Groningen, University of Groningen, Groningen, the Netherlands; 2 Department of Medical Microbiology and Infection Prevention, University Medical Center Groningen, University of Groningen, Groningen, the Netherlands; 3 Department of Medical Epidemiology, Certe Medical Diagnostics and Advice Foundation, Groningen, the Netherlands; 4 Data Science Center in Health, University Medical Center Groningen, University of Groningen, Groningen, the Netherlands; 5 Nivel, Netherlands Institute for Health Services Research, Utrecht, the Netherlands; 6 Department of General Practice, Erasmus Medical Center, Rotterdam, the Netherlands; 7 Department of Primary and Community Care, Radboud Institute of Health Sciences, Radboud University Nijmegen Medical Center, Nijmegen, the Netherlands; 8 Department of Family Medicine, CAPHRI Care and Public Health Research Institute, Maastricht University Medical Center, Maastricht, the Netherlands; 9 Tranzo, Department of Social and Behavioral Sciences, Tilburg University, Tilburg, the Netherlands; 10 Midwifery Science, AVAG, Amsterdam Public Health, Amsterdam University Medical Center, Vrije Universiteit Amsterdam, Amsterdam, the Netherlands

**Keywords:** general practice, patient acceptance of health care, health policy, COVID-19

## Abstract

**Background:**

Many countries observed a sharp decline in the use of general practice services after the outbreak of the COVID-19 pandemic. However, research has not yet considered how changes in healthcare consumption varied among regions with the same restrictive measures but different COVID-19 prevalence.

**Aim:**

To investigate how the COVID-19 pandemic affected healthcare consumption in Dutch general practice during 2020 and 2021, among regions with known heterogeneity in COVID-19 prevalence, from a pre-pandemic baseline in 2019.

**Design & setting:**

Population-based cohort study using electronic health records. The study was undertaken in Dutch general practices involved in regional research networks.

**Method:**

An interrupted time-series analysis of changes in healthcare consumption from before to during the pandemic was performed. Descriptive statistics were used on the number of potential COVID-19-related contacts, reason for contact, and type of contact.

**Results:**

The study covered 3 595 802 contacts (425 639 patients), 3 506 637 contacts (433 340 patients), and 4 105 413 contacts (434 872 patients) in 2019, 2020, and 2021, respectively. Time-series analysis revealed a significant decrease in healthcare consumption after the outbreak of the pandemic. Despite interregional heterogeneity in COVID-19 prevalence, healthcare consumption decreased comparably over time in the three regions, before rebounding to a level significantly higher than baseline in 2021. Physical consultations transitioned to phone or digital over time.

**Conclusion:**

Healthcare consumption decreased irrespective of the regional prevalence of COVID-19 from the start of the pandemic, with the Delta variant triggering a further decrease. Overall, changes in care consumption appeared to reflect contextual factors and societal restrictions rather than infection rates.

## How this fits in

It is known that the outbreak of the COVID-19 pandemic caused a sharp decrease in GP healthcare consumption. This study provides insights in how healthcare-seeking behaviour in general practice care differs among regions with the same restrictive measures but different COVID-19 prevalence in the Netherlands. This study indicates that changes in care consumption appeared to reflect contextual factors and societal restrictions rather than infection rates. This emphasises the importance of taking the impact of restrictive measures on healthcare-seeking behaviour into consideration when making policy decisions.

## Introduction

The outbreak of the COVID-19 pandemic had a major impact on all levels of health care.^1–3^ To reduce the pressure on hospital occupancy and ensure the availability of intensive care beds, most governments introduced restrictive measures designed to reduce COVID-19 transmission. Furthermore, there was a sharp decrease in healthcare consultations in general practice from the start of the pandemic,^4^ with evidence that many patients feared becoming infected with COVID-19 or being a burden on the healthcare system.^5,6^ Patients also experienced a real decrease in access to GPs owing to the restrictive measures imposed by practices, such as stricter triage and healthcare postponement.^7^ The pandemic therefore led to major organisational changes in general practice and differences in healthcare provision.^2,8,9^ This change was especially problematic in the Dutch healthcare system, where limited clinical capacity represents a more severe bottleneck than in other Western European countries.^10,11^

Considerable regional differences in COVID-19 prevalence were seen in the Netherlands during the early stages of the pandemic.^12^ The outbreak started in the South, with a peak prevalence in February 2020, before it spread to the East and North over the next 4 weeks. Despite this, the prevalence remained highest in the South, with a lower prevalence in the East and an even lower prevalence in the North. Consequently, when restrictive measures were imposed or eased nationwide, this was done regardless of different prevalence in these regions. We hypothesised that this would cause one of two patterns of healthcare consumption. On the one hand, consumption could decrease asynchronously in the three regions, following the epidemic’s evolution rather than the timeline of the imposed measures. On the other hand, it could decrease synchronously, implying the avoidance of potentially important health care owing to the nationally imposed measures instead of the local epidemiology.

In this study, we aimed to assess the impact of the COVID-19 pandemic on GP healthcare consumption in daily practice in the Netherlands, focusing on the number of consultations, potential COVID-19-related contacts, the reasons for contact, and the type of contacts among three regions. This study provides insights into how healthcare-seeking behaviour in general practice care differed among regions in the Netherlands, all subjected to the same restrictive measures but with varying COVID-19 prevalence rates after the outbreak of the pandemic. By examining these differences, we elucidate the influence of contextual factors and societal restrictions on healthcare-seeking behaviour, emphasising the importance of considering the impact of such measures when making policy decisions.

## Method

### Study design and data sources

In this population-based cohort, we used data from the electronic health records (EHRs) of general practices taking part in three GP research networks managed by university medical centres: the Academic General Practitioner Development Network (AHON), the Family Medicine Network (FaMe-Net), and the Research Network Family Medicine (RNFM). AHON is managed by the University Medical Center Groningen and includes 59 practices in the North of the Netherlands, FaMe-Net is managed by the Radboud University Medical Center Nijmegen and includes six practices in the East, and RNFM is managed by the Maastricht University Medical Center and includes 28 practices in the South. The Northern network (AHON) represented a region with a low prevalence at the start of the pandemic, while the Eastern network (FaMe-Net) represented an intermediate prevalence region, and the Southern network (RNFM) represented a high prevalence region. We included EHR data from the period between 1 January 2019 and 31 December 2021.

The study was performed according to the REporting of studies Conducted using Observational Routinely collected health Data statement.^13^ Data from the registry databases were pseudonymised to make individual patient details, and thereby removing the need for ethical approval according to Dutch law.^14^ Before starting this study, members from the patient association Zorgbelang Groningen were asked for their input on research design and researchers have reported back regularly about the conduct of this study.

### Data collection

Patients were eligible for inclusion if they were registered with a general practice affiliated to one of the included networks for at least one-quarter of a year during the observation period. Each network maintains a data registry of pseudonymised clinical data from the EHRs of patient contacts in general practice, including the contact type, clinical findings, diagnoses, and test results. EHR data from the three networks were pseudonymised before extraction. Death or deregistration from one of the included general practices ended patient eligibility. Contact data were linked to population data by use of pseudonyms. To ensure data quality, we excluded patients with an unknown date of birth, a date of birth before 1 January 1919, reason for deregistration without deregistration date, or missing data that prevented pseudonymisation (for example, postal code).

A healthcare contact was defined as a physical consultation (in clinic or at home), telephone consultation, or any form of online communication. Contacts were included if they took place between a patient and a GP or practice nurse, and if they recorded at least one International Classification of Primary Care (ICPC)-1 code with a note in the EHR (that is, the ‘journal’).^15^ Contacts could contain >1 ICPC-1 code (for example, when patients discussed multiple problems). Journals and contact types for included patients were extracted and matched to represent a contact. Duplicates that appeared during data extraction were removed. Finally, data on urbanisation and socioeconomic status were linked to data from Statistics Netherlands (Centraal Bureau voor de Statistiek) using pseudonyms.^16^

### Outcome measures

The primary outcome was healthcare consumption, defined as the number of GP contacts per 1000 registered patients per studied region. Secondary outcomes included the number of potential COVID-19-related contacts, reasons for each contact, and the type of contact (regular, phone, home, or digital consultation).

In the context of potential COVID-19-related contacts, we compiled a comprehensive list of all ICPC-1 codes that were deemed potentially linked to acute COVID-19 infection (see Supplementary Table S1). This list was initially constructed by drawing on previous research findings.^17^ However, recognising the evolving nature of our understanding of COVID-19 and its associated symptoms during the pandemic, we refined this ICPC list based on ongoing knowledge during the pandemic. To ensure the validity of the extended list, a GP (Maarten Homburg) and a microbiologist (Matthjis Berends) independently assessed the relevance of each ICPC-1 code to acute COVID-19 infection.

A potential COVID-19-related contact was then defined as a contact containing a relevant ICPC-1 code. For the analysis of reasons for contact, we clustered all contacts by ICPC-1 code into three categories using a previously published method: acute care (for example, K75, acute myocardial infarction); prolonged reversible care (for example, F92, cataract); and chronic irreversible care (for example, T90, diabetes).^18^

### Data analysis

The population characteristics and number of contacts are described in absolute numbers, as a percentage of the total population, and as medians and interquartile ranges (IQRs) by study year and demographic region. All contact types were aggregated and visualised as a proportion of total contacts per week in 2019, 2020, and 2021. We then analysed the number of contacts per 1000 patients per week and compared these with data for 2019, using a centred moving average of 5 weeks as the baseline in our visualisation. Differences in healthcare consumption from before to during the pandemic were compared using an interrupted time-series model for each of the three demographic regions.

To analyse interregional differences and maintain comparability, six pandemic phases were defined that reflected both the incidence of COVID-19 and the restrictive measures imposed by the Dutch Government. The phases were then modelled after the lowest and highest national infection rates, according to data from the National Institute for Public Health and the Environment (Table 1).^19^ For example, phase 1a covered the start of the pandemic to the peak national infection rate in week 15, before turning to phase 1b, which covered the period to gradual relaxation of restrictive measures (start of phase 2).

**Table 1.** COVID-19 pandemic phases in the Netherlands based on infection rates and restrictive measures

Phase of the COVID-19 pandemic in 2020 and 2021		Description of COVID-19 infection rates and containment measures
**2020**	**Phase 0** *week 1–8*	- No confirmed COVID-19 infections in the Netherlands
	**Phase 1** *week 9–22*	- First wave of COVID-19 infections - First lockdown (that is, promotion of good hand hygiene, social distancing, working at home, and schools, restaurants, amusement industry closed) - Highest infection rates: week 15
	**Phase 2** *week 23–40*	- Decrease in infection rates - Gradual relaxation of restrictive measures - Lowest infection rates: week 28
**2020–2021**	**Phase 3** *2020 week 40* *to* *2021 week 3*	- Second wave of COVID-19 infections - Stricter containment measures, start of a partial lockdown followed by a strict lockdown (curfew, and schools, stores, and sport facilities closed) - Highest infection rates: weeks 44 and 52
**2021**	**Phase 4** *week 4–16*	- Emergence of the Alpha variant (B1.1.7) of concern, further increase in infection rates, and continued lockdown measures - Highest infection rates: week 16
	**Phase 5** *week 17–43*	- Decrease in infection rates - Gradual opening up of society and only minor restrictions - Lowest infection rates: week 26
	**Phase 6** *week 44–52*	- Epidemic rise with the Delta variant (B.1.617.2) of concern and a steep increase in infection rates - Lockdown measures reintroduced - Highest infection rates: week 48

Regarding the time-series analysis, β-coefficients, with the accompanying standard error (SE), were used to estimate differences in intercept (that is, the number of contacts) and time after intercept (that is, how fast the number of contacts changes over time). Correction for seasonal effects was done by adding a seasonal intercept to the regression model. *P*-values <0.05 for the intercept or time after intercept during the phases were considered significant. All data were analysed using R (version 4.1.1) software.^20^

## Results

### Population characteristics

The study populations comprised 425 639 patients (3 595 802 contacts), 433 340 patients (3 506 637 contacts), and 434 872 patients (4 105 413 contacts) for 2019, 2020, and 2021, respectively (see Supplementary Figures S1a and S1b). Overall, 61% of patients were from the Northern region, 10% were from the Eastern region, and 29% were from the Southern region. Patients in the Northern region had median ages of 47 years (IQR 40) in 2019, 46 years (IQR 40) in 2020, and 45 years (IQR 40) in 2021. In the Eastern region, the median ages were 38 years (IQR 35) in 2019, 37 years (IQR 35) in 2020, and 37 years (IQR 35) in 2021. In the Southern region, the median ages were 49 years (IQR 39) in 2019, 48 years (IQR 38) in 2020, and 48 years (IQR 39) in 2021. As shown in Table 2, half of the patients were female, urbanisation was lowest in the Northern region and highest in the Southern region, and the regions had comparable distributions of socioeconomic statuses. Throughout the study period, the demographic and socioeconomic parameters remained relatively stable within each region, allowing us to assess the changes in healthcare-seeking behaviour per region while accounting for the inherent differences among the regions.

**Table 2.** Characteristics of included patients registered with GPs by study region and year

	North (**UMCG**), *n* (%)			East (**RUMC**), *n* (%)			South (**MUMC**), *n* (%)		
**Characteristic**	2019	2020	2021	2019	2020	2021	2019	2020	2021
**Population**	261 789 (100)	267 219 (100)	265 084 (100)	41 100 (100)	42 015 (100)	40 591 (100)	122 750 (100)	124 106 (100)	129 197 (100)
**Sex**									
Female	131 543 (50)	134 258 (50)	133 111 (50)	20 791 (51)	21 243 (51)	20 488 (50)	62 356 (51)	62 971 (51)	65 437 (51)
Male	130 246 (50)	132 961 (50)	131 973 (50)	20 309 (49)	20 772 (49)	20 103 (50)	60 394 (49)	61 135 (49)	63 760 (49)
**Age, years**									
0–4	5923 (2)	8249 (3)	10 769 (4)	1688 (4)	2365 (6)	2424 (6)	2657 (2)	3765 (3)	5200 (4)
5–14	26 394 (10)	26 822 (10)	26 539 (10)	5672 (14)	5651 (13)	5509 (14)	10 746 (9)	10 910 (9)	11 477 (9)
15–24	32 147 (12)	33 320 (12)	33 788 (13)	5127 (12)	5236 (12)	5061 (12)	13 138 (11)	13 316 (11)	13 964 (11)
25–44	58 576 (22)	60 086 (22)	58 905 (22)	12 319 (30)	12 662 (30)	12 073 (30)	28 830 (23)	29 149 (23)	29 387 (23)
45–64	72 987 (28)	74 034 (28)	73 181 (28)	10 713 (26)	10 694 (25)	10 437 (26)	34 818 (28)	35 132 (28)	36 763 (28)
65–84	55 427 (21)	55 278 (21)	53 873 (20)	4935 (12)	4852 (12)	4634 (11)	27 557 (22)	27 344 (22)	28 576 (22)
≥85	10 335 (4)	9430 (4)	8029 (3)	646 (2)	555 (1)	453 (1)	5004 (4)	4490 (4)	3830 (3)
**Number of contacts per patient**									
0	56 027 (21)	63 459 (24)	50 217 (19)	7499 (18)	9012 (21)	7069 (17)	32 475 (26)	33 831 (27)	24 217 (19)
1–5	83 920 (32)	87 366 (33)	86 001 (32)	18 933 (46)	19 738 (47)	19 057 (47)	45 497 (37)	45 390 (37)	47 823 (37)
6–10	43 708 (17)	42 485 (16)	45 054 (17)	7259 (18)	6927 (16)	7507 (18)	19 353 (16)	19 373 (16)	22 489 (17)
>10	78 134 (30)	73 909 (28)	83 812 (32)	7409 (18)	6338 (15)	6958 (17)	25 425 (21)	25 512 (21)	34 668 (27)
**Urbanisation^a,b^**									
Very high	12 741 (5)	13 799 (5)	15 096 (6)	4362 (11)	4314 (10)	3948 (10)	8149 (7)	8361 (7)	8183 (6)
High	26 858 (10)	27 399 (10)	27 109 (10)	7263 (18)	7084 (17)	6677 (16)	51 720 (42)	52 022 (42)	54 670 (42)
Average	47 563 (18)	48 455 (18)	60 879 (23)	16 183 (39)	16 829 (40)	16 635 (41)	16 216 (13)	17 028 (14)	19 244 (15)
Low	70 113 (27)	71 425 (27)	60 747 (23)	9776 (24)	10 407 (25)	10 415 (26)	22 402 (18)	22 604 (18)	24 764 (19)
Very low	87 078 (33)	87 954 (33)	88 488 (33)	915 (2)	810 (2)	688 (2)	12 893 (11)	13 081 (11)	14 747 (11)
Missing	17 436 (7)	18 187 (7)	12 765 (5)	2601 (6)	2571 (6)	2228 (5)	11 370 (9)	11 010 (9)	7589 (6)
**Socioeconomic Status^a,c^**									
Low	87 432 (33)	87 822 (33)	89 329 (34)	11 636 (28)	11 592 (28)	11 204 (28)	39 656 (32)	40 486 (33)	44 175 (34)
Middle	111 052 (42)	113 786 (43)	114 756 (43)	16 488 (40)	17 142 (41)	16 996 (42)	48 469 (39)	48 648 (39)	52 553 (41)
High	40 078 (15)	41 345 (15)	42 000 (16)	9340 (23)	9727 (23)	9785 (24)	19 559 (16)	20 153 (16)	21 473 (17)
Missing	23 227 (9)	24 266 (9)	18 999 (7)	3636 (9)	3554 (8)	2606 (6)	15 066 (12)	14 819 (12)	10 996 (9)

^a^Data on urbanisation and socioeconomic status (SES) is derived from Statistics Netherlands (CBS). Not all included patients could be linked to CBS data after pseudonymisation. ^b^Urbanisation: very high: ≥2500 addresses/km^2^; high: 1500–2499 addresses/km^2^; average: 1000–1499 addresses/km^2^; low: 500–999 addresses/km^2^; and very low: <500 addresses/km^2^. ^c^SES: based on standardised household income in the Netherlands: low: 0–40 percentile; middle: 41–80 percentile; and high: 81–100 percentile. MUMC = Maastricht University Medical Center. RUMC = Radboud University Medical Center Nijmegen. UMCG = University Medical Center Groningen.

### Changes in care consumption

**Figure 1.**
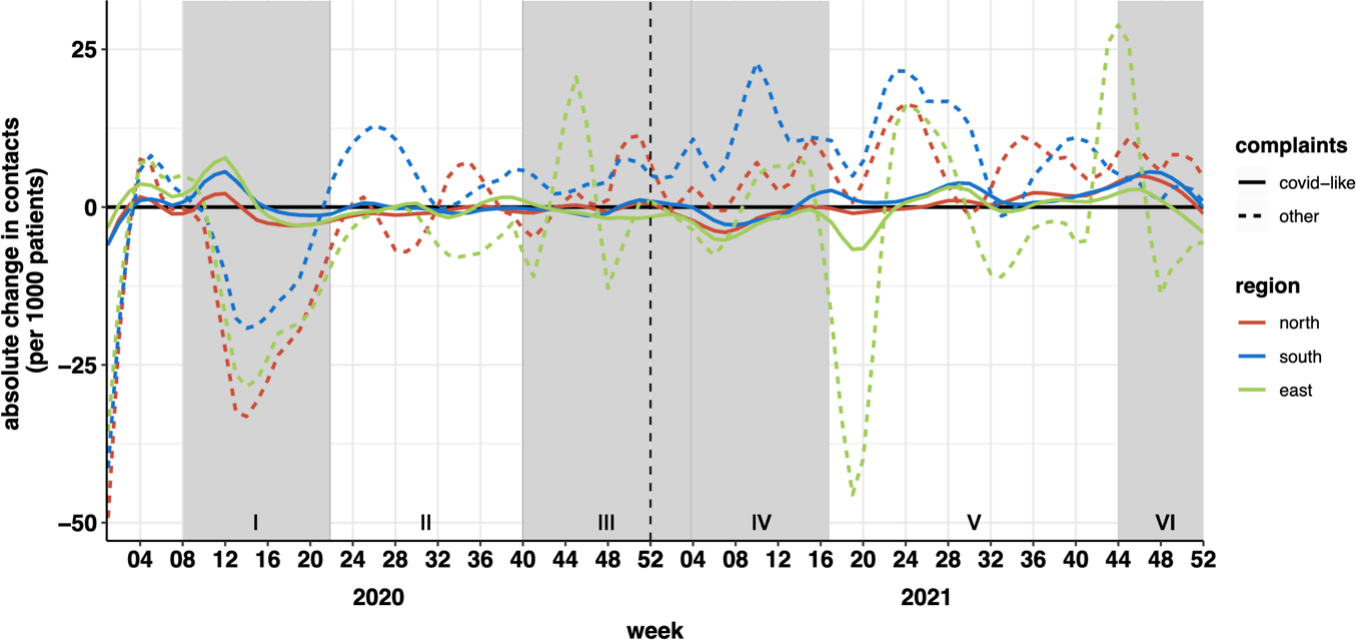
Changes in contacts from before to during the pandemic. The chart shows absolute change in contacts for COVID-19 and other complaints in 2020 and 2021 compared with 2019 by study region (University Medical Center Groningen, North; Radboud University Medical Center Nijmegen, East; and Maastricht University Medical Center, South). Grey shading represents periods of increased restrictions and rising COVID-19 infection rates. The sudden decrease in contacts in the Eastern region in week 19 of 2021 represents a failure in data extraction and not the true number of contacts.

Figure 1 visually illustrates healthcare consumption in the three regions relative to the pre-pandemic baseline. During the initial pandemic wave, there was a noticeable decline in overall healthcare consumption, and this trend emerged simultaneously in all three regions, despite the pandemic initially spreading from the Southern region to the Northern region. In this initial phase, potential COVID-19-related contacts increased, particularly in the Eastern and Southern regions, while the Northern region exhibited a relatively smaller rise in such contacts.

To evaluate the dynamics of healthcare consumption across the three distinct regions, a time-series analysis was conducted (Table 3). This analysis involved examining intercept values, which signify the healthcare consumption level at the onset of each phase, and time after intercept (TAI) values, which indicate the trend in healthcare consumption throughout each phase.

**Table 3.** Time-series analysis of care consumption for the different phases of the pandemic compared with pre-pandemic of patients registered with their GPs localised in the North (UMCG), East (RUMC), and South (MUMC) of the Netherlands in 2019, 2020, and 2021

	North (**UMCG**)		East (**RUMC**)		South (**MUMC**)	
**Mean number of contacts per week per 1000 patients, pre-pandemic**	**107.18**		**91.52**		**91.21**	
**Time (SE**)	–0.07 (0.1)		0.02 (0.11)		–0.09 (0.09)	
	**Intercept (SE**)	**TAI (SE**)	**Intercept (SE**)	**TAI (SE**)	**Intercept (SE**)	**TAI (SE**)
Phase 1a	9.88 (8.92)	**–6.86 (2.24)^a^**	12.85 (9.91)	**–5.74 (2.49)^a^**	8.07 (8.12)	–2.58 (2.04)
Phase 1b	**–28.05 (9.84)^a^**	1.14 (2.19)	**–24.12 (10.93)^a^**	0.09 (2.44)	–10.72 (8.96)	0.59 (2)
Phase 2a	6.57 (10.01)	–0.07 (2.77)	–5.44 (11.12)	1.12 (3.08)	14.90 (9.11)	0.91 (2.52)
Phase 2b	–5.67 (9.16)	1.24 (1.06)	–5.74 (10.18)	–0.59 (1.18)	10.50 (8.34)	–0.47 (0.97)
Phase 3a	–2.21 (11.48)	1.37 (5.19)	–5.43 (12.75)	–5.12 (5.76)	11.48 (10.44)	–3.41 (4.72)
Phase 3b	6.92 (10.08)	1.29 (1.93)	**37.88 (11.19)^a^**	**–8.01 (2.14)^a^**	9.77 (9.17)	0.45 (1.75)
Phase 3c	–13.60 (12.34)	9.67 (5.19)	–20.26 (13.71)	6.28 (5.76)	–10.72 (11.23)	**11.79 (4.72)^a^**
Phase 4	8.32 (10.03)	0.80 (0.99)	–11.84 (11.14)	1.79 (1.10)	16.38 (9.13)	1.01 (0.90)
Phase 5a	–4.02 (13.35)	**3.91 (1.41)^a^**	**–52.71 (14.83)^a^**	**8.15 (1.57)^a^**	11.72 (12.15)	**2.65 (1.29)^a^**
Phase 5b	10.21 (12.44)	0.42 (0.67)	–0.60 (13.82)	–0.62 (0.74)	**23.99 (11.32)^a^**	–0.67 (0.61)
Phase 6a	25.86 (14.54)	–1.63 (3.67)	**71.06 (16.15)^a^**	**–24.02 (4.08)^a^**	21.96 (13.23)	–0.81 (3.34)
Phase 6b	**31.26 (15.77)^a^**	**–13.03 (5.19)^a^**	6.37 (17.52)	**–12.62 (5.76)^a^**	25.16 (14.35)	**–11.01 (4.72)^a^**
**Summer**	8.02 (4.07)		1.41 (4.52)		0.21 (3.7)	
**Autumn**	–3.25 (3.87)		–4.72 (4.3)		–6.12 (3.53)	
**Winter**	6.44 (3.87)		7.51 (4.3)		1.57 (3.52)	
**R^2^**	0.47		0.61		0.40	
**Adjusted R^2^**	0.35		0.52		0.27	
**Residual SE**	11.60 (df = 127)		12.88 (df = 127)		10.55 (df = 127)	
**F statistic**	3.97^a^ (df = 28; 127)		7.09^a^ (df = 28; 127)		3.05^a^ (df = 28; 127)	

^a^ *P*<0.05. df = degrees of freedom. MUMC = Maastricht University Medical Center. RUMC = Radboud University Medical Center Nijmegen. SE = standard error. TAI = time after intercept. UMCG = University Medical Center Groningen.

At the onset of the pandemic (phase 1a), intercept values across all regions were comparable with the pre-pandemic baseline in 2019. From this moment, there was a significant negative trend in TAI values for the Northern and Eastern regions. This rapid decline in healthcare consumption, as indicated by TAI values of –6.86 (North) and –5.74 (East), suggested a swift drop in GP consultations and healthcare-seeking behaviour shortly after the pandemic began. The Southern region also exhibited a negative TAI trend, albeit less pronounced.

In phase 1b, coinciding with the peak infection rates (week 15), both the Northern and Eastern regions experienced substantial decreases in healthcare consumption, as evidenced by their negative intercept values (North: –28.05, East: –24.12, both *P*<0.05). In contrast, the South, which saw a less pronounced TAI trend during phase 1a, displayed a less significant decrease in consultations.

Phase 2, which ran from week 23 to week 40 of 2020, saw healthcare consumption in all regions return to levels comparable with the pre-pandemic baseline.

Phase 3, corresponding to the second wave of COVID-19 infections, displayed notable regional variation in healthcare consumption. The Northern region maintained a stable pattern, while the Eastern and Southern regions experienced peaks in consultations in weeks 44 and 49, respectively.

Phase 4 (weeks 4–16, 2021) exhibited relatively consistent healthcare consumption patterns across the three regions, with no significant deviations from the pre-pandemic baseline. This period coincided with the emergence of the Alpha variant of concern (B1.1.7) and the continuation of certain lockdown measures.

Phase 5 marked a transition with the gradual relaxation of pandemic-related restrictions. During this phase, all regions experienced a significant rising trend in GP consultations, as indicated by significant positive TAI values, reflecting heightened demand for GP services among the population.

Conversely, phase 6 coincided with the reintroduction of lockdown measures in response to the epidemic rise with the Delta variant (B.1.617.2) of concern. In this phase, all three regions witnessed a significant decrease in healthcare consumption, as highlighted by their respective negative TAI values, emphasising the immediate impact of stricter measures on healthcare utilisation patterns.

Seasonal variations in healthcare utilisation observed in 2019 did not significantly differ from those observed during the subsequent years of the COVID-19 pandemic. Additionally, the Eastern region experienced a sudden excessive decrease in contacts during week 19 of 2021. However, this corresponded with a brief interruption in data extraction from the research network, which temporarily impacted the completeness of our dataset for that particular week. While this extraction issue was promptly identified, thorough checks were conducted to ensure that the remaining data were reliable and accurate.

### Reason for contact

**Figure 2.**
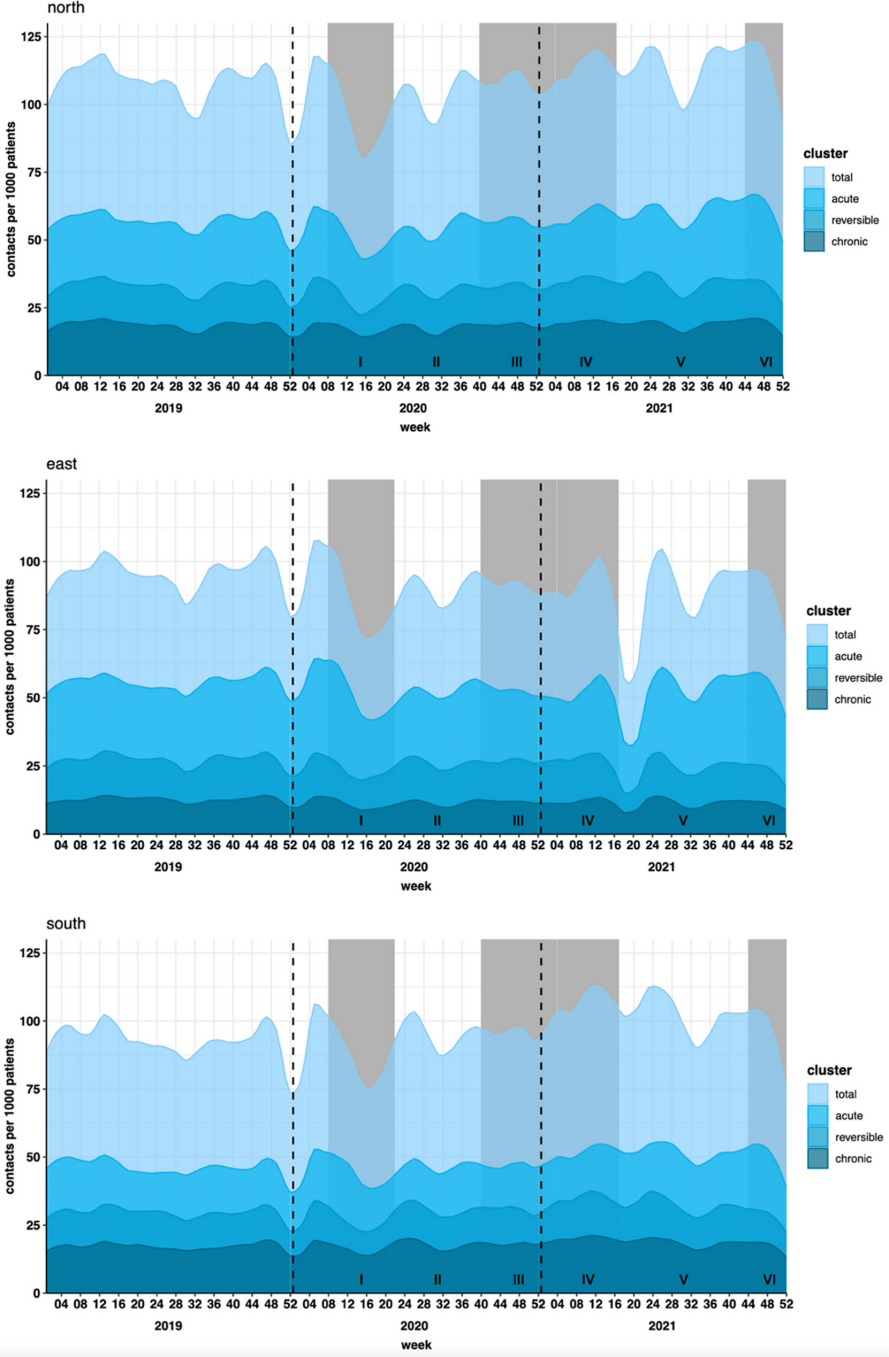
Type of healthcare consumption by study region and year. Healthcare consumption is shown per 1000 patients for acute, prolonged (reversible), and chronic (irreversible) illness by study region (University Medical Center Groningen, North; Radboud University Medical Center Nijmegen, East; and Maastricht University Medical Center, South) and year (2019, 2020, and 2021). Grey shading represents periods of increased restrictions and rising COVID-19 infection rates

The pandemic was associated with fluctuations in demand for acute care, prolonged reversible care, and chronic irreversible care (Figure 2). These seemed to reflect the overall changes in healthcare consumption, with all three clusters showing comparable patterns of decreasing and increasing trends in each of the regions (Figure 2 and Supplementary Table S2).

### Type of contact

**Figure 3.**
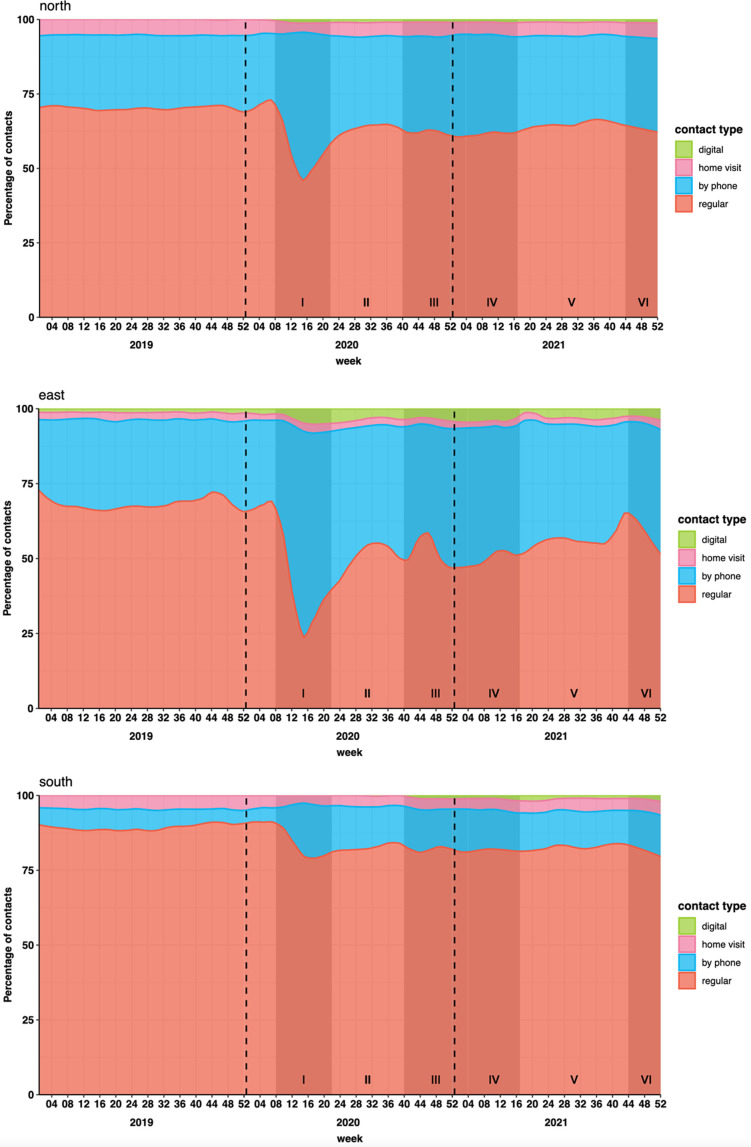
Type of healthcare contact by study region and year. Contact types are shown by study region (University Medical Center Groningen, North; Radboud University Medical Center Nijmegen, East; and Maastricht University Medical Center, South) and year (2019, 2020, and 2021). Shading represents periods of increased restrictions and rising COVID-19 infection rates.

After the start of the pandemic, the proportion of physical contacts decreased sharply among the three regions and the proportion of phone contacts increased sharply. All three studied regions showed this transition in consult type from immediately after the start of the first lockdown in phase 1. As the pandemic continued, the proportions of physical consults increased, but they did not reach pre-pandemic levels again. All three studied regions showed minimal use of digital consultations in 2019, with use still increasing by the end of 2022 (Figure 3).

## Discussion

### Summary

The key finding of this research is that healthcare consumption decreased simultaneously in regions with high and low COVID-19 infection rates after the outbreak of the pandemic. Moreover, while the implementation of social restrictions during the COVID-19 pandemic may have contributed to reduced infectious disease transmission, and therefore fewer healthcare contacts related to infections, we observed similar trends in healthcare consumption across all types of care, including acute, chronic, and prolonged illnesses (Figure 2). This suggests that the effects of lockdowns extended beyond their potential impact on infectious diseases, influencing healthcare-seeking behaviour on a broader scale.

Potential COVID-19 contacts also increased in all regions, although the greatest increases were seen in the Eastern and Southern regions, consistent with the spread of the epidemic.^12^ The early stage of the pandemic was also associated with a decrease in the proportion of in-person contacts and an increase in remote care. During the second and subsequent phases, all demographic regions experienced increased proportions of in-person contacts, although these remained below pre-pandemic levels.

After the initial decrease in healthcare consumption at the start of the pandemic, time-series analysis showed healthcare consumption returning to levels comparable with the pre-pandemic baseline in phase 2, indicating a temporary return to normal GP consultations.

Phase 3 exhibited notable regional variation in healthcare consumption, with the Northern region maintaining stability and the Eastern and Southern regions experiencing peaks in consultations in weeks 44 and 49, respectively. These peaks can largely be attributed to the high attendance of the yearly influenza and pneumococcal vaccination campaigns in 2020, typically taking place during this period.^21^

After relatively consistent healthcare consumption patterns during phase 4, in phase 5 there was a significant transition with the gradual relaxation of pandemic-related restrictions. During this phase, all regions experienced a noteworthy rising trend in GP consultations, reflecting heightened demand for GP services among the population. This increase might represent a compensation for the loss of care observed earlier in the pandemic.

Conversely, with the reintroduction of restrictive measures in phase 6, all three regions witnessed a significant decrease in healthcare consumption again, emphasising the immediate impact of stricter measures on healthcare utilisation patterns.

These findings highlight the dynamic nature of healthcare consumption patterns during the COVID-19 pandemic and emphasise the importance of considering the broader implications of restrictive measures on healthcare-seeking behaviour. Despite no explicit government recommendations to avoid seeking health care, the data clearly illustrate how increasing and decreasing of restrictive measures have a pronounced effect on healthcare consumption, underscoring the importance of clear communication during public health crises. Future pandemic preparedness efforts should aim to mitigate potential negative consequences on general population health while addressing the challenges posed by infectious disease outbreaks.

### Strengths and limitations

This study demonstrates how harmonised data from different GP research networks in the Netherlands can be used to assess regional differences in healthcare consumption in areas that differed in COVID-19 prevalence after the outbreak of the pandemic. We included large and diverse cohorts representative of the Dutch population in each studied region. Furthermore, our data are enhanced by data linkage between EHRs and Statistics Netherlands to provide insights about the urbanisation and socioeconomic statuses of the included patients.

It should be noted that EHR data are not intended for scientific research. Consequently, our best efforts aside, limited registration may have resulted in some GP activities being missed (for example, digital consultations). This may have hindered our measurements of healthcare consumption. However, we did attempt to overcome this limitation with the use of thorough data cleaning and by working closely with data managers in each region to identify potential limitations in their data.

While our study provides valuable insights into healthcare consumption patterns within the selected regions, it does not cover the entirety of the Netherlands, and there may be variations in healthcare-seeking behaviour in regions not included in our analysis.

### Comparison with existing literature

Our findings of an initial sharp decrease in consultations and a transition from regular to phone and digital consultations are consistent with international trends.^4,6,22^ Researchers have also reported that one in five people avoided healthcare services during lockdowns^6,23^ and that patients experienced decreased access to primary health care throughout the pandemic (for example, stricter triage made it harder to get an appointment).^7,24–27^ Indeed, these findings might explain the observed decrease in healthcare consumption during lockdown in the present study, although whether this will have a long-term effect on disease outcomes remains to be confirmed. Research has also shown that people avoided health care for potentially life-threatening illness (for example, cardiovascular disease, stroke, and malignancy),^28–31^ as indicated by the initial decrease in acute, prolonged, and chronic healthcare consumption in the current research.

While our study primarily concentrated on healthcare consumption in daily general practice settings, it is noteworthy that similar trends in healthcare consumption for both potential COVID-19-related contacts and other healthcare contacts were observed in GP out-of-hours services in the Netherlands during the first year of the pandemic.^17^ Given the gatekeeper function of the GP in the Netherlands, where patients contact their GP or GP out-of-hours services first before receiving care at a hospital or emergency department, the observed trends emphasise the broader impact of the pandemic on health-seeking behaviour across various healthcare settings. These findings highlight the pivotal role of GPs in coordinating patient care during a public health crisis.

Although the potential consequences of healthcare avoidance are clear for life-threatening symptoms, decreasing follow-up opportunities or postponing chronic care could lead to increased hospitalisations, and eventually, increased mortality.^32,33^

### Implications for research and practice

The findings of this study have considerable clinical implications. Our results show that the COVID-19 pandemic had a large impact on care consumption in Dutch general practice. Changes were seen in both the number of consultations and the provision of care in this setting, although these appear to be more closely related to contextual factors (for example, lockdown measures or fear of contracting COVID-19) than on the actual regional epidemiology. This might explain the observed healthcare avoidance and reduced access in general practice, particularly when lockdown measures were introduced in regions with low infection rates.

The findings underscore the need for healthcare providers, including GPs, to be aware of the nuanced dynamics of patient behaviour during pandemics. It emphasises the importance of clear and effective patient communication strategies after the outbreak of a major epidemic to encourage appropriate healthcare utilisation. Future research could focus on patient trajectories after avoiding care for acute, prolonged, or chronic diseases, aiming to gain insights into the consequences for both patients' health and future healthcare consumption. Additionally, attention should be given to the types of consultations offered, especially in the context of the transition to telephone or digital consultations.

Overall, we recommend that the potential effect of restrictive measures on health-seeking behaviour should be considered when making policy decisions because healthcare avoidance and the potential for missed diagnosis could have serious consequences for both individual patients and wider society.

## Data

Access to the data used in this study can be facilitated after obtaining approval from the steering committees of the involved GP networks. The dataset employed in our research consists of registration texts from GPs, detailing sensitive and confidential information about patients' health. However, it's important to note that the dataset is not freely available; permission must be sought, along with potential associated fees, before access is granted. These arrangements are in place to ensure proper data usage. For more information about the dataset's specifics as used in this study, enquiries can be directed to the corresponding author.
